# Endoscopic transcecal appendectomy for a laterally spreading tumor of the appendiceal stump

**DOI:** 10.1055/a-2512-3926

**Published:** 2025-01-31

**Authors:** Xin-Yue Li, Dan-Feng Zhang, Meng-Jiang He, Quan-Lin Li, Ping-Hong Zhou

**Affiliations:** 1 92323 Endoscopy Center and Endoscopy Research Institute, Zhongshan Hospital Fudan University, Shanghai, China; 2 Shanghai Collaborative Innovation Center of Endoscopy, Shanghai, China

A 68-year-old woman, who was diagnosed with suppurative appendicitis 17 years previously and underwent appendectomy, was found during a recent colonoscopy, performed as part of a health-check, to have a 1.5-cm flat laterally spreading tumor (LST) at the appendiceal orifice ( Fig. 1 **a** ). Biopsy indicated that the lesion was a tubular adenoma with low grade intraepithelial neoplasia (LGIN). Computed tomography revealed peristump exudation and pneumatosis. Endoscopic transcecal appendectomy (ETA) was recommended to remove the lesion at the appendiceal stump ( Video 1 ).

**Fig. 1:**
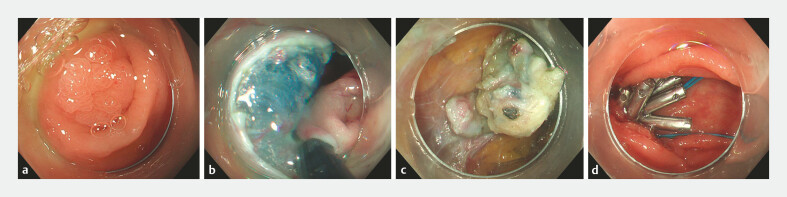
Endoscopic images showing: **a** a laterally spreading tumor at the appendiceal orifice; **b** full-thickness resection around the lesion; **c** dissection of the appendiceal stump in the peritoneal cavity; **d** closure of the cecal defect using the purse-string suture technique.

Endoscopic transcecal appendectomy is performed for a laterally spreading tumor of the appendiceal stump.Video 1

After submucosal injection had been performed and a circumferential incision made using a HookKnife, an IT Knife was used to perform a full-thickness resection around the lesion ( Fig. 1 **b** ). The colonoscope was then introduced into the peritoneal cavity, where extensive tissue adhesions were seen, and we thoroughly dissected the appendiceal stump from the surrounding fibrous tissue ( Fig. 1 **c** ). The appendiceal stump and the lesion were completely resected and extracted through the anus using a snare ( Fig. 2 **a** ). After the wound had been cauterized with hot biopsy forceps and it had been confirmed that there was no active bleeding, the cecal wall defect was closed with 12 endoclips and a nylon loop ( Fig. 1 **d** ). A nasogastric tube was fixed in the cecum for anal decompression using dental floss and an endoclip. The total procedure duration was 60 minutes. The patient was discharged on postoperative day 6, without having experienced any adverse events. Subsequent pathologic diagnosis confirmed chronic stump appendicitis and a localized tubular adenoma with LGIN ( Fig. 2 **b** ).

**Fig. 2:**
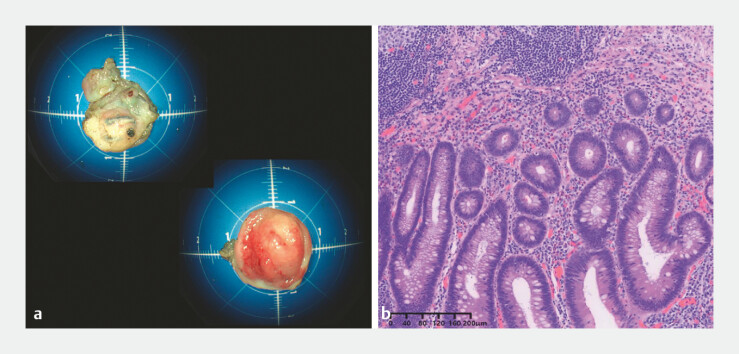
Pathology of the resected specimens showed: **a** macroscopically, complete resection of the appendiceal stump and lesion (the base and the apex); **b** microscopically, chronic stump appendicitis and a localized tubular adenoma with low grade intraepithelial neoplasia.

ETA is a natural orifice transluminal endoscopic surgery, which has proved to be safe and effective for appendiceal orifice lesions 1 . In this case, ETA was advantageous because of the presence of peritoneal adhesions from the prior surgery, as it facilitated control during resection and avoided the need for a second surgical intervention. Recently, we have also reported a similar case of ETA for stump appendicitis 2 , and clinical studies in larger cohorts with long-term follow-up are warranted to evaluate the safety and efficacy of ETA in patients who have previously undergone appendectomy.

Endoscopy_UCTN_Code_TTT_1AQ_2AD_3AF

## Notes

Endoscopy E-Videos https://eref.thieme.de/e-videos
*E-Videos* is an open access online section of the journal *Endoscopy* , reporting on interesting cases and new techniques in gastroenterological endoscopy. All papers include a high-quality video and are published with a Creative Commons CC-BY license. Endoscopy E-Videos qualify for HINARI discounts and waivers and eligibility is automatically checked during the submission process. We grant 100% waivers to articles whose corresponding authors are based in Group A countries and 50% waivers to those who are based in Group B countries as classified by Research4Life (see: https://www.research4life.org/access/eligibility/ ). This section has its own submission website at https://mc.manuscriptcentral.com/e-videos .
